# Nurses’ practical contributions to improving healthy and sustainable public spaces: an integrative review

**DOI:** 10.1590/0034-7167-2024-0023

**Published:** 2025-01-10

**Authors:** Herlys Rafael Pereira do Nascimento, Ingrid Mikaela Moreira de Oliveira, Grayce Alencar Albuquerque, Edilma Gomes Rocha Cavalcante, Marlene Menezes de Souza Teixeira, Maria do Socorro Vieira Lopes

**Affiliations:** I Universidade Regional do Cariri. Crato, Ceará, Brazil; II Universidade Estadual do Ceará. Fortaleza, Ceará, Brazil

**Keywords:** Health Knowledge, Attitudes, Practice, Healthy Lifestyle, Nurses, Nurses, Community Health, Sustainable Development, Conocimientos, Actitudes y Práctica en Salud, Ciudad Saludable, Enfermeras y Enfermeros, Enfermeros de Salud Comunitaria, Desarrollo Sostenible

## Abstract

**Objective:**

to identify knowledge production about nurses’ contributions to improving healthy and sustainable public spaces.

**Methods:**

an integrative review carried out in February 2023 in electronic databases. Studies that answered the research question and that were available in full, in Portuguese, English and Spanish, were included.

**Results:**

a total of five articles were selected. The findings highlighted the importance of educational projects in the training of local managers and community autonomy; citizen participation and health promotion as ways to implement Sustainable Development Goal 11; nurses as facilitators of collective care; new health practices and modes of producing subjectivity; and use of public transportation, bicycles and/or walking in these spaces.

**Final considerations:**

there is a clear need for greater incentives from local governments to develop effective sustainability strategies that are led by nurses and the community.

## RESUMEN

**Objetivo:**

identificar la producción de conocimiento sobre las contribuciones de enfermeros para la mejora de espacios públicos saludables y sostenibles.

**Métodos:**

revisión integradora, realizada en febrero de 2023 en bases de datos electrónicas. Se incluyeron publicaciones que respondieron a la pregunta de investigación y que estaban disponibles en su totalidad en portugués, inglés y español.

**Resultados:**

se seleccionaron cinco artículos en total. Los hallazgos señalaron la importancia de los proyectos educativos en la formación de gestores locales y la autonomía comunitaria; participación ciudadana y promoción de la salud como formas de implementar el Objetivo de Desarrollo Sostenible 11; el enfermero como facilitador del cuidado colectivo; nuevas prácticas de salud y modos de producción de subjetividad; y uso del transporte público, bicicleta y/o caminar en estos espacios.

**Consideraciones finales:**

se necesitan mayores incentivos por parte de los gobiernos locales para desarrollar estrategias de sostenibilidad efectivas que sean dirigidas por enfermeros dentro de la comunidad.

## INTRODUCTION

The first technical standard for sustainable cities in Brazil was approved by the *Associação Brasileira de Normas Técnicas* (ABNT, Brazilian Association of Technical Standards), receiving the name of *Norma Brasileira Regulamentadora* (NBR, Brazilian Regulatory Standard), from the International Organization for Standardization (ISO) 37.120/2017. This standard defines and establishes methodologies for a set of indicators related to the sustainable development of urban communities, with the aim of guiding and measuring urban services’ performance and quality of life in cities^(1)^.

There are currently 16 urban agglomerations in Brazil, with more than 1 million inhabitants each, led by the megacities of São Paulo and Rio de Janeiro, but followed by several others. Such population concentration, without the corresponding growth in the supply of physical infrastructure (housing, sanitation, public transport), social infrastructure (education, health, leisure), employment and income, leads part of the population to live in precarious conditions in slums or other forms of settlement, where misery, human degradation and organized crime proliferate; the latter bringing violence closer to the people^(2)^.

Several actions have been taken to address inequities and the problems caused by the concentration of population in cities, such as the emergence and implementation of social agendas, such as Healthy Cities. Today, due to our government’s commitment to the Sustainable Development Goals (SDGs) and the United Nations (UN) agenda that incorporates them, such as Sustainable Cities, it has as its backdrop the concepts of promoting health, equity and sustainability^(3)^.

The Healthy Cities initiative, encouraged by the World Health Organization (WHO) and its counterpart in the Americas, the Pan American Health Organization (PAHO), has been an essential strategy for improving populations’ quality of life. In addition to recognizing health in its positivity as an expression of quality of life, a city is expected to healthily generate participatory, social and institutional processes in the collective elaboration of a certain vision of the city and, above all, seek to agree on a collective and targeted intervention in all social policies towards one goal, which is to continually improve the lives of all citizens^(4)^. This macro understanding, therefore, emphasizes not only the notion of process, but of shared commitment. A city does not become healthy by decree, as the notion of a long and continuous process, decision and political will permeate all individual and collective work.

Today, the European Healthy Cities Network still exists, but the Canadian network has been losing strength over time. In Latin America, the Latin American Healthy Cities Network continues to exist, being strongest in Mexico, Argentina and Chile. Brazil participates in this network through the movement of potentially healthy municipalities and with the Northeastern network of healthy municipalities^(4)^.

During the Rio+20 Conference, which took place in the city of Rio de Janeiro (2012), the discussion process began to expand and resolve some problems that the Millennium Development Goals (MDGs) had not given much emphasis to: adoption of a set of global objectives that would meet the planet’s social, economic and environmental integration. In 2015, at the United Nations Conference on Sustainable Development, the 2030 Agenda and the SDGs, which would replace the MDGs, were unanimously approved by the organization’s member countries. In the same year, two international conferences also marked the discussion process for SDG implementation: Conference of the Parties (COP21), with the approval of the Paris Agreement to reduce the increase in global temperature on the planet; and the III International Conference on Development, in Addis Ababa, Ethiopia, which established financing flows for policies with economic, social and environmental priorities^(5)^.

The United Nations Climate Change Conference (COP-26), held from October 31 to November 12, 2021, one year late due to the pandemic that ravaged the world, hosted in Glasgow (Scotland), had the following objectives: a) seek to neutralize emissions of harmful gases, limiting global warming to 1.5˚C; b) protect the ecosystems of countries affected by climate change; c) obtain funds to finance these goals; and d) seek global cooperation between governments and civil society, regulating the Paris Agreement^(6)^.

In the 21^st^ century, the UN 2030 Agenda, adopted by world leaders in September 2015, lists 17 SDGs for building a sustainable reality, based on urgent, bold and transformative measures. The SDGs propose tasks for people and institutions, in all parts of the planet, to be accomplished by the year 2030. Among the SDGs, there is Goal 11, which corresponds to sustainable cities and communities, and States, institutions and other social actors must transform cities and human settlements into inclusive, safe, resilient and sustainable spaces^(7)^.

The concept of healthy and sustainable public spaces incorporates basic sanitation, clean and structurally adequate physical spaces, support networks to achieve healthy and safe psychosocial habits, free from violence of any kind^(2)^, as well as balanced interaction with the ecosystem, ensuring a safe environment for the current and future generations.

Thus, there is an urgent need for a transformative proposal for local knowledge and practices, through public policies that create projects within the demands of sustainable development and raising awareness among the population, through environmental education, to conceive a broad territorialization, attracting dynamics between inhabiting and experiencing the territory, considering the aspects of constructing the population’s living and health conditions, economically, socially, culturally and politically, making the territory viable as a category of social analysis^(8)^.

Health promotion, combined with environmental education, can be a proposal used to achieve such objectives, since, considering the social, economic and cultural diversity of people, it can configure the territory as a space of belonging and representation, where the principles of sustainability and well-being would be disseminated. Health promotion is understood as a set of strategies, policies, actions and interventions with the purpose of improving individuals’ and communities’ quality of life, as it acts on the social conditions and determinants of health, in an intersectoral manner and with popular participation, promoting healthy choices by individuals and communities in the territory where they are located, seeking to identify the means to control the factors that favor or hinder their well-being and that of the community, and avoiding risks of illness and harm to their quality of life^(9)^.

Health does not only mean treating illnesses, care and rehabilitation tasks. Caring for health means preventing illnesses and promoting contexts that are favorable to life and dignity. Health promotion is one of the areas of collective health that brings together knowledge and practices aimed at promoting social and environmental conditions for a healthy and dignified life within communities^(10)^.

Thus, nursing has much to contribute to reversing the cycle of environmental degradation and people becoming ill, since developing educational practices on the subject in question, carried out under the leadership of nurses, considering that these professionals work strongly in family health and in the context in which they live, emerges as a key point to disseminate actions for sustainability and reduction of unsafe, non-inclusive and non-resilient spaces. Therefore, environmental health must permeate all nursing activities, aiming at improving healthy and sustainable public spaces.

## OBJECTIVE

To identify knowledge production about nurses’ contributions to improving healthy and sustainable public spaces.

## METHOD

### Ethical aspects

Since this was a study that used public domain data and did not involve human beings, there was no need for assessment by a Research Ethics Committee. However, it is important to note that the studies selected for the final sample were duly referenced.

### Study design

This is an integrative literature review. PVO strategy was used to formulate the research question (P - population and/or problem situation; V - variables; O - outcome). The following structure was considered: P - nurses; V - health practices; O - healthy and sustainable public spaces. The following guiding question emerged: what practices are carried out by nurses to improve healthy and sustainable public spaces?

### Literature search

The search strategy was carried out through the *Coordenação de Aperfeiçoamento de Pessoal de Nível Superior* (CAPES, the Coordination for the Improvement of Higher Education Personnel) Journals Portal, Online System for Searching and Analysis of Medical Literature (MEDLINE/PubMed), *Literatura Latino-Americana e do Caribe em Ciências da Saúde* (LILACS), *Índice Bibliográfico Español en Ciencias de la Salud* (IBECS), Web of Science, and the Scientific Electronic Library Online (SciELO).

To reach studies on this topic, the “Healthy City”, “Nurses”, “Nursing”, “Health Knowledge, Attitudes, Practice” health descriptors (DeCS/MeSH) and the “Sustainable Development Goal 11” search term were used. Cross-referencing was performed in English, using the Boolean operators AND and OR, as can be seen in Chart 1:

**Chart 1:** Search strategies for each data source

MEDLINE/PubMed	((((nurses[Title]) OR (nursing[Title])) AND (Health Knowledge, Attitudes, Practice)) AND (Healthy City[Title/Abstract])) OR (Sustainable Development Goal 11[Title])
**SciELO**	(Nursing) OR (nurses) AND (Health Knowledge, Attitudes, Practice) AND (ti:(Healthy City)) OR (ti:(Sustainable Development Goal 11))
**LILACS and IBECS**	(ti:(Nurses)) OR (ti:(nursing)) AND (tw:(Health Knowledge, Attitudes, Practice)) AND (ti:(Healthy City)) OR (ti:(Sustainable Development Goal 11))
**Web of Science**	(((((TI=(“Nurses”)) OR TI=(“Nursing”)) AND AK=(“Health Knowledge, Attitudes, Practice”)) AND TI=(Healthy City^*^)) OR ALL=(Sustainable Development Goal 11)) AND PY=(2015-2022)

The search and selection of articles were carried out in February 2023.

### Article selection process

Full-text articles, available in full, without a time frame, in Portuguese, English and Spanish, and that brought practical contributions from nurses, were included. Articles that did not provide abstracts, editorials, theoretical reflections, experience reports, reviews, monographs, theses and dissertations, abstracts in conference proceedings and duplicate studies were excluded. In order to reduce possible errors or biases in study search and selection, it was proposed that these be carried out by two reviewers simultaneously and independently. It is worth noting that the article selection stage was developed with the help of EndNote^®^ Web reference manager, a free online version, for better standardization and greater potential for study reproducibility. The included studies underwent an analysis with the aim of organizing and summarizing extracted elements.

### Data extraction and categorization

Data extraction was performed using a structured form containing information on authorship, year, country, study objectives, design, main findings, level of evidence and degrees of recommendation^(11)^. A thorough analysis of the articles in full allowed us to recognize how the selected studies address the development of practices aimed at achieving SDG 11, which were organized into categories.

## RESULTS

The cross-referencing of the descriptors resulted in 2,379 studies. After reading the titles and abstracts of the studies found, 95 articles were selected for skimming, of which 11 were not available online and 50 did not meet the inclusion criteria, leaving 34 to be screened.

**Figure 1:**
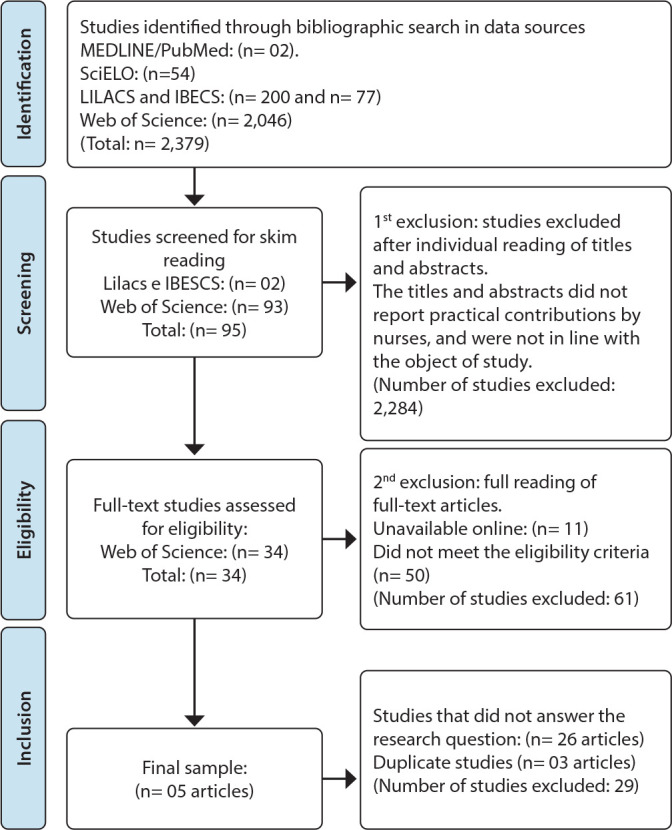
Flowchart representing the selection of studies analyzed in this research. Crato, Ceará, Brazil, 2023

The second stage of the selection process consisted of reading the 34 complete documents in full, of which 26 did not respond to the study’s question and three articles were duplicates. Thus, the final *corpus* consisted of a sample of five articles. These studies were saved in a folder using a code (e.g., A1, where A stands for article, and 1 is the order number). Figure 1 shows the article selection process flowchart.

Among the selected articles, there was a slight predominance of international studies, two from Brazil^(12,13)^, one from Slovenia^(14)^ and two from Spain^(15,16)^, with practical contributions from nurses related to SDG 11. The most prevalent language was English, present in four^(12,14-16)^ of the articles, and only one^(13)^ used Portuguese. The articles were published between 2011 and 2021, with a predominance in the last five years^(12,15,16)^. Although the SDGs emerged in 2015, a study^(13)^ published in 2011 brought notions that culminated in the thinking of SDG 11 by presenting the figure of the nurse as an important actor in the performance of collective care actions for individuals’ health. This reinforces the decision not to use a time frame in the study, because although the SDGs were published in 2015, the MDGs, prior to the SDGs, already addressed environmental issues.

All selected articles are indexed in Web of Science, with two published in the journal Sustainability^(14,16)^, one in Globalization and Health^(15)^, one in the International Journal of Health Promotion and Education^(12)^, and one in the *Revista Latino-Americana de Enfermagem* ^(13)^. Concerning study design, two are qualitative studies^(12,13)^, two are case studies^(14,15)^, and one is a descriptive study with quantitative analysis^(16)^.

The studies listed presented actions that can be developed within cities and communities, seeking to make them healthier and more sustainable spaces. Some studies present nurses as a relevant figure in these processes, however, other studies presented them as an integral part of the various characters involved in this endeavor, in which case their role is diluted among the strategies developed by the most varied sectors of society.

Therefore, all articles selected in this study address SDG11 implementation from a collective and territorial view of its application, highlighting the importance of the involvement of managers and professionals from different sectors and the most diverse characters in society in achieving this goal, which can be seen in Chart 2.

**Chart 2:** Characteristics of selected studies regarding authorship, year, country, study objective, design, main findings, level of evidence and degree of recommendation, 2023

Variables	Selected articles
**Article identification code**	**A1^(^** ^12)^
**Author/year/country**	Almeida ACL, Davey P. / (2020)/ Brazil.
**Article title**	Integrating health promotion into sustainable development goal 11: major challenges and learned lessons from Healthy Municipalities, Cities and Communities (HMC) in Brazil.
**Study objectives**	To examine how healthy cities and municipalities initiatives can contribute to integrating health promotion into SDG 11 implementation, creating synergies at local levels. To propose a framework for integrating health promotion into SDG 11 implementation at local level.
**Outline**	Qualitative research, using interviews as a source of data collection.
**Main findings**	Development of education and local development projects, focusing on training local managers and community autonomy. Social participation, as a pillar of health promotion and an essential tool for implementing SDG 11. Nurses are among the figures who work towards this end.
**Level of evidence/degree of recommendation**	4/C
**Article identification code**	**A2** ^(13)^
**Author/year/country**	Fortuna CN, Matumoto S, Pereira MJB, Mishima SM, Kawata LS, Camargo-Borges C./ (2011)/ Brazil
**Article title**	*O enfermeiro e as práticas de cuidados coletivos na estratégia saúde da família*
**Study objectives**	To identify and analyze the collective care practices of nurses in the family health strategy and their structuring knowledge.
**Outline**	Qualitative research, using semi-structured interviews as a source of data collection.
**Main findings**	Nurses are important actors in triggering collective care actions in family health, as they propose, organize, develop and evaluate such actions. The rupture with the dominant and established way of intervening in the health territory requires devices that question its logic and expose its functioning, denaturalizing and destabilizing its arrangements and meanings, thus enabling the construction of new health practices, new ways of producing subjectivity.
**Level of evidence/degree of recommendation**	4/C
**Article identification code**	**A3** ^(14)^
**Author/year/country**	Szander N, Ros-McDonell L, Fuente-Aragone MV, Vodopivec R./ (2018)/ Slovenia.
**Article title**	Sustainable Urban Homecare Delivery with Different Means of Transport
**Study objectives**	To compare different means of transportation in an urban environment as a crucial input for an algorithm for scheduling home care activities, with patient satisfaction as the top priority.
**Design**	Case study.
**Main findings**	Efficient and reliable routes that consider urban sustainability goals, reducing travel time and the working hours of nurses working in home care when using public transportation, bicycle and/or walking.
**Level of evidence**	4/C
**Article identification code**	**A4** ^(15)^
**Author/year/country**	Rubio OR, Daher C, Fanjul G, Gascon M, Mueller N, Pajín L, Plasencia A, Rueda DR, Thondoo M, Nieuwenhuijsen MJ/ (2019)/ Spain.
**Article title**	Urban health: an example of a “health in all policies” approach in the context of SDGs implementation.
**Study objectives**	To understand the links between social determinants of health, environmental exposures, behavior, health outcomes and urban policies within the SDGs, following a health in all policies (HiAP) logic; review and analyze the key elements of a HiAP approach as an accelerator of the SDGs in the context of urban and transport planning; and describe lessons learned from the practical implementation of integrated health actions in cities in Europe, Africa and Latin America.
**Design**	Case study with implementation of a strategic assessment tool.
**Main findings**	The analysis confirmed that the SDG framework offers an opportunity to formulate and implement policies with a HiAP approach. Three important aspects are highlighted: 1) the importance of intersectoral work and health equity as a cross-cutting issue in sustainable development actions, including interventions by health professionals such as nurses; 2) policy coherence, health governance and stakeholder participation as key issues; and 3) the need for high-quality data.
**Level of evidence/degree of recommendation**	4/C
**Article identification code**	**A5** ^(16)^
**Author/year/country**	Córdoba PJ, Esteban VA, Bento B./ (2021)/ Spain.
**Article title**	The Commitment of Spanish Local Governments to Sustainable Development Goal 11 from a Multivariate Perspective
**Study objectives**	To analyze Spanish local governments’ commitment to SDG-11, indicating possible improvements with the aim of fulfilling the 2030 Agenda.
**Design**	This is a descriptive study of a quantitative nature, using the X-STATIS study technique for data analysis.
**Main findings**	The results indicate a positive trend towards achieving SDG-11, in which progressive governments are concerned with issues of inclusive and sustainable urbanization as a result of greater citizen participation, and conservative governments are focusing on improving the safety of slums through inclusive and accessible public spaces. In this scenario, health professionals, such as nurses, appear to play a supporting role in the development of actions that contribute to inclusive and sustainable development.
**Level of evidence/degree of recommendation**	4/C

## DISCUSSION

Discussions about the impact of changes that have occurred in the environment have been more frequent in recent times, presenting evidence of their relevance for ecosystems, regardless of their size or scale. In the case of human beings, the scale of populations, the impact and magnitude of environmental changes caused by species have made this issue a priority in the search for preserving the planet’s survival^(17)^.

It is noticeable that the number of articles selected for this study was not very expressive, and this is due to the fact that there are few studies that contemplate contributions from nurses to improving healthy and sustainable public spaces, which may signal the urgent need for greater action by nursing in environmental health, considering that nurses work mainly in health promotion and disease prevention, and most of these involve environmental issues.

The following topics will be presented for this discussion, based on the articles included in this study, namely: 1) Development of education projects focusing on the training of local managers and community autonomy; 2) Social participation and health promotion for implementing Sustainable Development Goal 11; 3) Nurses as facilitators of collective care actions in family health; 4) Construction of new health practices and new modes of producing subjectivity; 5) Use of public transport, bicycles and/or walking in sustainable cities.

### 1) Development of education projects focusing on the training of local managers and community autonomy

Among the 17 goals, SDG 3 (Good health and well-being) is considered relevant to achieving other goals. SDG 11 (Sustainable cities and communities) is a key goal to be included at local levels with regard to achieving the SDGs. It was possible to identify that municipalities are developing local development education projects, with a focus on training local managers and community autonomy, for food security and health promotion, prevention of violence against women with a focus on social inclusion, female empowerment and self-esteem^(12)^. These actions are being developed mainly within Primary Health Care (PHC) services, in which nurses play a leading role in the organization and implementation of these projects.

However, the Federal Constitution of Brazil, in Article 182, established that a municipality is responsible for planning and executing urban policy. This establishes that master plans are mandatory for municipalities with more than 20,000 inhabitants^(18)^, as well as procedures for participatory urban planning, including approval by the City Council before implementation. In fact, by bringing together the main themes discussed by the community, Healthy Cities and Municipalities (HCM) initiatives direct local governments towards the inclusion of health promotion in participatory master plans^(12)^.

Thus, it is possible to conclude that the lack of support from the federal government hinders the widespread implementation of HCM in Brazil, and most likely hindered the availability of health promotion infrastructure to guarantee local managers the implementation of HCM projects, including the level of awareness, human resources and financing^(12)^. Likewise, there is still the obstacle due to the fact that, even though some health professionals recognize the convergence of health and well-being promotion in cities, public health is resistant to adopting health promotion as a guiding paradigm, and public health policies are still centered on diseases^(19)^.

In particular, local governments’ commitment to cities’ sustainable development is demonstrated by the upgrading of slums, increasing access to housing, providing adequate basic services to citizens (Adequate housing) and increasing sustainability in transport systems (Commuters). Furthermore, direct participation by civil society has increased in recent years (Citizen participation), as has concern for less developed countries, to which financial assistance has increased (Transfers). There was also an increase in the average proportion of paved surface in cities with open spaces for public use (public roads), which was accompanied by an improvement in air quality as well as a reduction in the levels of smaller diameter particles (air pollution) that cause most respiratory diseases, which, in general, improves urban life^(16)^.

However, there is still much to be done in relation to education for developing healthy and sustainable public spaces. Therefore, greater investment is needed by local governments to implement educational models, enabling better infrastructure and training of human resources to truly raise awareness in society as well as to achieve a supportive awareness that aims to preserve the public good and life as a whole.

### 2) Social participation and health promotion for implementing Sustainable Development Goal 11

Social participation and integration of SDGs into master plans have been identified as key factors that can promote integrated SDG 11 implementation at local levels^(12)^. As for the challenges to be overcome, the lack of a national strategy with general guidelines for implementation in municipalities supported by the federal government and the absence of assessment of the effectiveness of the results achieved by HCM were highlighted by stakeholders as the most important aspects that may hinder SDG implementation at the local level^(20-22)^.

According to the UN, SDG implementation should be based on a participatory approach^(23)^. Indeed, social participation is a main pillar of the HCM strategy, enhancing the process and changes at local levels^(21)^. HCM initiatives involve collaboration and participation of different actors and use of different participatory strategies. Furthermore, mobilization and collective participation in decision-making processes are principles of health promotion^(19)^. PHC can be one of the effective ways to disseminate to society these basic principles that unite health and sustainability, placing nurses and their teams as key players in this awareness-raising process.

Political will, community participation and training of these human resources are facilitating factors that make the implementation of HCM initiatives viable^(24)^, with community participation being an essential part of effective health promotion^(25)^. The adoption of participatory methods (e.g., participatory assessments, participatory planning, development of virtual networks, e-government tools and collaborative projects) and intersectoral actions in local projects and initiatives can strengthen community participation, facilitating a more integrated and efficient SDG 11 implementation at the local level^(12)^. PHCs, for instance, should receive more investment from local governments to create more participatory channels with the community, aiming at greater community integration in sustainable urban space development.

Progressive governments, in favor of increasing spending through debt, focus their efforts on inclusive and sustainable urbanization, based on greater citizen participation, which leads to constructing paved stretches and developing new residential areas^(16)^. On the other hand, conservative governments prioritize improvement of slums, safe, inclusive and accessible public spaces and the importance of daily collection of urban solid waste. Moreover, the ideology seems to be losing influence on other targets, such as those related to climate change^(16)^.

### 3) Nurses as facilitators of collective care actions in family health

Hegemonic care actions in providing health care lack strategies for change, as they are still focused on individuals, being curative and disconnected from real health needs^(26)^. From this theoretical perspective, the collective is considered a multiple composition of various connections, powers and ruptures^(27)^. They are flows of life and desire that intersect with dams that interrupt them; they are changing interconnections that are singularized in encounters and disagreements of affections, ways of living and values^(27)^.

For research purposes, collective health care actions were considered to be those commonly accepted in this field, such as the joint construction of the area’s diagnosis (territorialization process), health promotion activities, social participation and control, health education and intersectoral actions^(13)^.

In a study by Fortuna *et al*.^(13)^, a nurse said that collective care actions are those that are planned, assessed and discussed with the team, implementing them with the aim of: seeking acceptance and adherence from the population, and for them to have an easier life and quality of life, which would be to become sick less; having more opportunities, a more dignified life, a good job, good housing, basic hygiene instructions; planning life, not only in relation to children, but also in relation to expenses, understanding the fact that illnesses are not just what they seem; and having more access to information and general care that the unit can and should offer.

Although still incipient, in nurses’ statements, this perspective can be a booster of other ways of doing and knowing collective care, as it calls for a complexity for health needs and for the work process, for which interlocution of knowledge and co-responsibility among team, family and other sectors are necessary^(13)^.

Therefore, nurses have a prominent role to play in health services, especially at primary level, with regard to reflecting on new ways of providing health, in a more sustainable way and in harmony with the environment, incorporating, together with the multidisciplinary team, actions that make it possible to achieve SDG 11. Urban development has a significant impact on people’s physical and mental health and housing, and environmental problems can influence cities to become epicenters of non-communicable diseases, overloading health systems^(28,29)^.

### 4) Construction of new health practices and new modes of producing subjectivity

Unprecedented changes in recent decades have led to increased complexity of social structures, global health problems, and inequalities within and between nations. The challenges of climate change and epidemiological and demographic transitions leading to the rise of noncommunicable diseases and population aging require a rethinking of the way we develop public health policies^(30)^. Cities are home to more than half of the world’s population^(31)^, and the urban context offers an unprecedented opportunity to understand the links among health, its social determinants and the environment, and how to implement solutions following an intersectoral approach^(15)^.

A deeper understanding of the interconnections in how cities are designed, planned, built and governed and how this directly affects human health has evolved significantly in recent years. Two global milestones have driven the idea that local decision-making processes that recognize urban policies are, in fact, the main public health interventions. The first is the approval, in 2015, of the 2030 Agenda for Sustainable Development^(32)^, composed of 17 SDGs and 169 targets, with global geographic scope.

The second milestone occurred in 2016, with the recently adopted New Urban Agenda at Habitat III, the United Nations Conference on Housing and Sustainable Urban Development^(31)^. This was the first time that ‘health’ appeared as a cross-cutting issue and was explicitly recognized as a central component of urban planning and governance, in addition to the delivery of health services. WHO reinforced these links by gathering growing scientific evidence linking the quality of urban design and transport with a range of health outcomes^(33)^. For instance, there is enough scientific evidence to link lifestyle and eating habits to health outcomes such as obesity and diabetes^(34)^, air pollution to cardiovascular and respiratory diseases and cancers^(35)^, or noise pollution to mental health problems and cardiovascular diseases^(36)^.

Green and blue open spaces in and around cities are very important. Preserving watersheds reduces contamination of drinking water, saving on water purification costs. Recycling, reusing and reducing solid waste eliminates the need to burn or bury it, improving air quality and reducing water and soil contamination. Better wastewater and sewage management, in a context of rising temperatures and extreme weather events related to climate change, also improves public health by reducing exposure to water-borne and mosquito-borne diseases, such as the recent urban Zika and Chikungunya epidemics^(37)^.

WHO defines HiAP as “an approach to policymaking that systematically considers the health implications of decisions across sectors, seeking synergies and avoiding harmful health effects of policies outside the health sector in order to improve both population health and health equity”^(15,37)^. The HiAP strategy provides strong and effective “horizontal governance”^(38)^, with an approach to complex health problems that involves the highest levels of government, political and executive leadership, leading to effective priority setting, innovation in policy formulation and implementation of sustainable solutions^(15)^.

Finally, a key aspect of implementing the urban health SDGs is ensuring that the commitment to leaving no one behind is translated into effective action. This requires an accurate understanding of the target populations, their needs and circumstances, and no one better than the nurse to act in this perspective, as well as through health education, empowering the population in relation to these environmental and health issues for the future of the next generations. It is also important that the available information is disaggregated according to the main axes of inequality, such as social class, gender, age or ethnicity/migration, using appropriate tools and metrics that should be widely available and include the Urban Health Index, which provides information on health inequalities in small areas within cities, or the Urban Health Equity Assessment and Response Tool^(39)^, which measures and takes action to address inequalities.

However, the information needed to measure socioeconomic inequalities efficiently (exposure or outcome data by different social groups at the small area level or, ideally, georeferenced) is often not available, particularly in settings with lower resources.

### 5) Use of public transport, bicycles and/or walking in sustainable cities

SDG 11 refers to harmful elements such as air pollution, but also to positive environmental exposures such as open green spaces. SDG 11.2 refers to “access to safe, affordable, accessible, and sustainable transport systems for all”, notably expanding public transport, and, although not explicitly mentioned in the SDGs, promoting cycling and walking^(15)^. The adoption of these forms of transport can be encouraged by health professionals, especially nurses, through health education in nursing consultations, encouraging healthier habits that will support truly sustainable public spaces.

These elements are also mutually dependent and therefore susceptible to change as a consequence of urban planning and transport policies and interventions. Indeed, a growing body of scientific evidence on urban policies’ health impacts can clarify risks and inform decision-making for sustainable development^(40)^. Healthy urban policies can significantly reduce infectious and non-communicable diseases and improve well-being. For instance, compact urban design capitalizes on population density to reduce greenhouse gas emissions and improve mobility, walkability and social cohesion, and thus health and well-being^(41)^.

Efficient public transport, in combination with cycling networks, promotes more physical activity, reducing air pollution and reducing overall traffic deaths and injuries^(42)^. Health outcomes are good drivers of policies in other sectors that may be unpopular, such as traffic restrictions or speed limits in cities. Several biases are commonly present in urban and transport planning, such as in the area of mobility, where most interventions are based on the needs and perspectives of healthy, wealthy and male people^(43,44)^. Participatory processes are needed to identify inclusive priorities for vulnerable subpopulations (women, older adults, people with disabilities).

Since the predominant use of motor vehicles in cities will continue to have a strong environmental and socioeconomic impact^(45)^, the increasing pressure on urban realities has led to a much greater interest in all aspects of sustainability in urban planning, development and all types of transport (goods, services and people). There is currently a great deal of concern about developing policies, programs and projects that take sustainability into account^(46)^.

A study noted the reduction in total working hours of nurses per day by combining bus and walking, showing that nurses who ride electric bicycles reduced, respectively, about 35% and 44% of the total time that nurses would spend commuting. This result is applicable to an urban environment, where the public transportation network is sufficient and cycling is allowed on a reasonable number of roads^(14)^. Better management of home care can support efficient use of healthcare resources, high-quality home care, and aspirations for livable communities and sustainable development. There is currently a general trend towards making urban services more sustainable, especially in denser urban areas.

### Study limitations

In this review, five articles were included, due to the scarcity of research carried out in this area. Taking into account that the theme of SDG 11, although current and of great relevance for cities and urban communities, requires greater incentives for researchers in environmental health to carry out studies, it aims to change paradigms and create educational proposals that redefine the lifestyles of the population not only in the country, but across the entire planet. It is recommended that new studies be carried out with designs that have a higher level of evidence.

### Contributions to nursing, health or public policy

This study provides a deep reflection on the actions in which nurses can be a strong ally within the multidisciplinary team, developing them with the purpose of improving or transforming public spaces into healthier and more sustainable environments, as this is one of the goals of the 2030 Agenda, with society, as a whole, involved in this process. In this regard, nurses appear as great facilitators of practices that contribute to this scenario, and they need to recognize this leadership space that they have in order to be able to initiate strategies that make this transformation possible. Logically, nurses will not be alone in this endeavor, as they must rely on intersectoral and governmental partnerships to achieve this much-desired goal.

## FINAL CONSIDERATIONS

To develop the actions listed in this study, nurses need intersectoral support and greater investment from local governments to be able to develop tools for promoting education and care, for real sustainability, in order to encourage individual involvement in the educational process, contribute to citizen participation and develop the autonomy of those involved in the process of building healthy spaces. The aim is to systematically use daily professional experiences through problem-based education, established from a dialogical and participatory perspective. It is expected that, based on a reality guided by critical reflection, transformative action will be achieved.
