# Supplementary material for: Nurses’ practical contributions to improving healthy and sustainable public spaces: an integrative review
Source: Rev Bras Enferm. 2025 Jan 10;77(Suppl 2):e20240023. doi: 10.1590/0034-7167-2024-0023 (PMC11726809; doi:10.1590/0034-7167-2024-0023)
Supplement: Translation

# Translation: Contribuições práticas de enfermeiros para a melhoria de espaços públicos saudáveis e sustentáveis: revisão integrativa

**DOI:** 10.1590/0034-7167-2024-0023pt

**Authors:** Herlys Rafael Pereira do Nascimento, Ingrid Mikaela Moreira de Oliveira, Grayce Alencar Albuquerque, Edilma Gomes Rocha Cavalcante, Marlene Menezes de Souza Teixeira, Maria do Socorro Vieira Lopes

**Affiliations:** I Universidade Regional do Cariri. Crato, Ceará, Brasil; II Universidade Estadual do Ceará. Fortaleza, Ceará, Brasil

**Keywords:** Conhecimentos, Atitudes, Práticas em Saúde, Cidade Saudável, Enfermeiras e Enfermeiros, Enfermeiros de Saúde Comunitária, Desenvolvimento Sustentável

## Abstract

**Objetivo:**

identificar a produção de conhecimento sobre as contribuições dos enfermeiros para a melhoria de espaços públicos saudáveis e sustentáveis.

**Métodos:**

revisão integrativa, realizada em fevereiro de 2023 em bases de dados eletrônicas. Foram incluídas publicações que respondessem à questão de pesquisa e que estivessem disponíveis na íntegra nos idiomas português, inglês e espanhol.

**Resultados:**

foram selecionados cinco artigos ao total. Os achados apontaram para a importância de projetos de educação na formação de gestores locais e na autonomia comunitária; participação cidadã e promoção da saúde como formas de implementar o Objetivo de Desenvolvimento Sustentável 11; o enfermeiro como facilitador do cuidado coletivo; novas práticas de saúde e modos de produção de subjetividade; e uso de transporte público, bicicleta e/ou caminhada nestes espaços.

**Considerações Finais:**

percebe-se a necessidade de maiores incentivos dos governos locais para o desenvolvimento de estratégias eficazes à sustentabilidade que sejam lideradas pelos enfermeiros junto à comunidade.

## INTRODUÇÃO

A primeira norma técnica para cidades sustentáveis do Brasil foi aprovada pela Associação Brasileira de Normas Técnicas (ABNT), recebendo o nome de Norma Brasileira Regulamentadora (NBR), da *International Organization for Standardization* (ISO) 37.120/2017. Essa norma define e estabelece metodologias para um conjunto de indicadores relacionados ao desenvolvimento sustentável de comunidades urbanas, com o objetivo de orientar e medir o desempenho de serviços urbanos e qualidade de vida nas cidades^(1)^.

Existem, hoje, no Brasil, 16 aglomerações urbanas, com mais de 1 milhão de habitantes cada, lideradas pelas megametrópoles de São Paulo e Rio de Janeiro, mas seguidas por várias outras. Tal concentração populacional, sem o correspondente crescimento da oferta de infraestrutura física (moradias, saneamento, transporte público), infraestrutura social (educação, saúde, lazer), emprego e renda, leva parte da população a viver em condições precárias em favelas ou outras formas de assentamento, onde proliferam a miséria, a degradação humana e o crime organizado; esse último trazendo para perto das pessoas a violência^(2)^.

Várias ações têm sido feitas para o enfrentamento das iniquidades e os problemas acarretados pela concentração de população nas cidades, como o surgimento e implementação de agendas sociais, como a Cidades Saudáveis. Hoje, em função do compromisso do nosso governo com os Objetivos de Desenvolvimento Sustentável (ODS) e com a agenda da Organização das Nações Unidas (ONU) que os incorpora, como a Cidades Sustentáveis, possui como pano de fundo as concepções de promoção da saúde, equidade e sustentabilidade^(3)^.

A iniciativa Cidades Saudáveis, estimulada pela Organização Mundial da Saúde (OMS) e pela sua correspondente nas Américas, a Organização Pan-Americana da Saúde (OPAS), tem sido uma estratégia essencial para a melhoria da qualidade de vida das populações. Além de reconhecer a saúde em sua positividade como expressão de qualidade de vida, espera-se de uma cidade que, de forma saudável, gere processos participativos, sociais e institucionais na elaboração coletiva de uma determinada visão de cidade e, sobretudo, busque pactuar uma intervenção coletiva e direcionada a todas as políticas sociais para um objetivo, que é melhorar continuamente a vida de todos os cidadãos^(4)^. Essa macro compreensão, portanto, enfatiza não só a noção de processo, mas de compromisso compartilhado. Uma cidade não se torna saudável por decreto, pois a noção de processo longo e contínuo, decisão e vontade política perpassam todo o trabalho individual e coletivo.

Hoje, a Rede Europeia de Cidades Saudáveis ainda existe, mas a canadense foi perdendo força ao longo do tempo. Na América Latina, a Rede Latino-Americana de Cidades Saudáveis continua existindo, sendo mais forte no México, Argentina e Chile. O Brasil participa dessa rede através do movimento dos municípios potencialmente saudáveis e com a rede nordestina de municípios saudáveis^(4)^.

Durante a Conferência Rio+20, que ocorreu na cidade do Rio de Janeiro (2012), iniciou-se o processo de discussão para ampliar e resolver alguns problemas que os Objetivos de Desenvolvimento do Milênio (ODM) não tinham dado tanto destaque: adoção de um conjunto de objetivos globais que atendessem à integração social, econômica e ambiental do planeta. Em 2015, na Conferência das Nações Unidas sobre Desenvolvimento Sustentável, foram aprovados, por unanimidade, pelos países-membro da organização, a Agenda 2030 e os ODS, que substituiriam os ODM. No mesmo ano, duas conferências internacionais também marcaram o processo de discussão para a implementação dos ODS: Conferência das Partes (COP21), com a aprovação do Acordo de Paris para reduzir o aumento da temperatura global do planeta; e a III Conferência Internacional para o Desenvolvimento, em Adis Abeba, Etiópia, que estabeleceu fluxos de financiamento para políticas com prioridades econômicas, sociais e ambientais^(5)^.

A Conferência das Nações Unidas sobre Mudanças Climáticas (COP-26), realizada de 31 de outubro a 12 de novembro de 2021, com um ano de atraso em função da pandemia que assolou o mundo, sediada em Glasgow (Escócia), teve como objetivos: a) buscar neutralizar as emissões de gases nocivos, limitando o aquecimento global a 1,5˚C; b) proteger os ecossistemas dos países afetados pelas mudanças climáticas; c) obter fundos para financiar essas metas; e d) buscar uma cooperação global entre governos e a sociedade civil, regulamentando o Acordo de Paris^(6)^.

No século XXI, a Agenda 2030 da ONU, adotada pelas lideranças mundiais em setembro de 2015, enumera 17 ODS, para a construção de uma realidade sustentável, a partir de medidas urgentes, ousadas e transformadoras. Os ODS propõem tarefas para pessoas e instituições, em todas as partes do planeta, a serem cumpridas até o ano de 2030. Entre os ODS, existe o Objetivo 11, que corresponde às cidades e comunidades sustentáveis, sendo que é dever dos Estados, das instituições e dos demais atores sociais transformar cidades e assentamentos humanos em espaços inclusivos, seguros, resilientes e sustentáveis^(7)^.

O conceito de espaços públicos saudáveis e sustentáveis incorpora saneamento básico, espaços físicos limpos e estruturalmente adequados, redes de apoio para se obter hábitos psicossociais são e seguros, isentos de violência de qualquer natureza^(2)^, bem como interação com o ecossistema de forma equilibrada, garantindo um ambiente seguro para a geração atual e as futuras.

Dessa forma, existe a urgência de uma proposta transformadora de saberes e práticas locais, através de políticas públicas que criem projetos dentro das demandas de desenvolvimento sustentável e sensibilização da população, por meio da educação ambiental, para conceber uma territorialização de forma ampla, atraindo dinâmicas entre o habitar e o vivenciar o território, diante das vertentes de construção da condição de vida e saúde da população, economicamente, socialmente, culturalmente e politicamente viabilizando o território como uma categoria de análise social^(8)^.

A promoção da saúde, aliada à educação ambiental, pode ser uma proposta utilizada para o alcance de tais objetivos, pois, considerando a diversidade social, econômica e cultural das pessoas, pode configurar o território como um espaço de pertença e representatividade, onde seriam difundidos os princípios de sustentabilidade e bem-estar. Entende-se por promoção da saúde um conjunto de estratégias, políticas, ações e intervenções com o propósito de melhorar a qualidade de vida dos indivíduos e coletividades, na medida em que atua sobre os condicionantes e determinantes sociais da saúde, de forma intersetorial e com participação popular, promovendo escolhas saudáveis por parte dos indivíduos e coletividades no território onde estão inseridos, buscando identificar os meios para conseguir controlar os fatores que favorecem ou dificultam seu bem-estar e o da comunidade, e evitando riscos de adoecimento e prejuízos para a sua qualidade de vida^(9)^.

A saúde não significa apenas tratar das doenças, tarefas da assistência e da reabilitação. Cuidar da saúde significa prevenir doenças e promover contextos favoráveis à vida e à dignidade. A promoção da saúde é uma das áreas da saúde coletiva que reúne conhecimentos e práticas voltados a promover as condições sociais e ambientais para uma vida saudável e digna no âmbito das coletividades^(10)^.

Dessa forma, a enfermagem tem muito a contribuir para reverter o ciclo de degradação ambiental e adoecimento das pessoas, pois o desenvolvimento de práticas educativas acerca da temática em questão, realizadas sob a liderança do enfermeiro, considerando que esse profissional atua fortemente na saúde das famílias e no contexto onde essas vivem, surge como ponto-chave para a disseminação das ações de sustentabilidade e redução de espaços inseguros, não inclusivos e não resilientes. Portanto, a saúde ambiental deve permear toda a atuação do enfermeiro, visando a melhoria de espaços públicos saudáveis e sustentáveis.

## OBJETIVO

Identificar a produção de conhecimento sobre as contribuições dos enfermeiros para a melhoria de espaços públicos saudáveis e sustentáveis.

## MÉTODO

### Aspectos éticos

Por se tratar de estudo que utilizou dados de domínio público e não envolveu seres humanos, não houve necessidade de apreciação pelo Comitê de Ética em Pesquisa. Contudo, ressalta-se que os estudos selecionados para a amostra final foram devidamente referenciados.

### Delineamento da pesquisa

Trata-se de revisão integrativa da literatura. Para a formulação da questão de pesquisa, foi utilizada a estratégia PVO (P - população e/ou situação problema; V - variáveis; O - *outcome*/desfecho). Considerou-se a seguinte estrutura: P - enfermeiros; V- práticas em saúde; O - espaços públicos saudáveis e sustentáveis. Surgiu a seguinte pergunta norteadora: quais as práticas realizadas por enfermeiros para a melhoria de espaços públicos saudáveis e sustentáveis?

### Busca na literatura

A estratégia de busca foi realizada por meio do Portal de Periódicos da Coordenação de Aperfeiçoamento de Pessoal de Nível Superior (CAPES), nas fontes de dados Sistema *Online* de Busca e Análise de Literatura Médica (MEDLINE/PubMed), Literatura Latino-Americana e do Caribe em Ciências da Saúde (LILACS), *Índice Bibliográfico Español en Ciencias de la Salud (IBECS), Web of Science e Scientific Electronic Library Online (SciELO).*

Para o alcance das publicações acerca dessa temática, utilizaram-se os descritores de saúde (DeCS/MeSH) *“Healthy City”, “Nurses”, “Nursing”, “Health Knowledge, Attitudes, Practice”* e o termo de busca “*Sustainable Development Goal 11*”. Os cruzamentos foram realizados em língua estrangeira, idioma inglês, com o uso dos operadores booleanos *AND* e *OR*, como pôde-se observar no Quadro 1.

**Quadro 1:** Estratégias de busca para cada fonte de dados

MEDLINE/PubMed	*((((nurses[Title]) OR (nursing[Title])) AND (Health Knowledge, Attitudes, Practice)) AND (Healthy City[Title/Abstract])) OR (Sustainable Development Goal 11[Title])*
**SciELO**	*(Nursing) OR (nurses) AND (Health Knowledge, Attitudes, Practice) AND (ti:(Healthy City)) OR (ti:(Sustainable Development Goal 11))*
**LILACS e IBECS**	*(ti:(Nurses)) OR (ti:(nursing)) AND (tw:(Health Knowledge, Attitudes, Practice)) AND (ti:(Healthy City)) OR (ti:(Sustainable Development Goal 11))*
***Web of Science***	(((((TI=(“Nurses”)) OR TI=(“Nursing”)) AND AK=(“Health Knowledge, Attitudes, Practice”)) AND TI=(Healthy City^*^)) OR ALL=(Sustainable Development Goal 11)) AND PY=(2015-2022)

A busca e a seleção dos artigos foram realizadas em fevereiro de 2023.

### Processo de seleção dos artigos

Foram incluídos artigos completos, disponíveis na íntegra, sem recorte temporal, nos idiomas português, inglês e espanhol, e que trouxessem contribuições práticas de enfermeiros. Foram excluídos artigos que não disponibilizassem resumos, editoriais, reflexões teóricas, relatos de experiência, resenhas, monografias, teses e dissertações, resumos em anais de eventos e estudos duplicados. Para que houvesse a redução de possíveis erros ou vieses na busca e seleção dos estudos, propôs-se que as mesmas fossem realizadas por dois revisores, de forma simultânea e independente. Ressalta-se que a etapa de seleção dos artigos foi desenvolvida com o auxílio do gerenciador de referências *EndNote* ^®^ *Web*, versão *online* e gratuita, para uma melhor uniformização e maior potencial de reprodutibilidade do estudo. Os trabalhos incluídos passaram por uma análise, com o objetivo de organizar e sumarizar os elementos extraídos.

### Extração e categorização dos dados

A extração dos dados foi realizada a partir de formulário estruturado contendo autoria, ano, país, objetivos do estudo, delineamento, principais achados, nível de evidência e graus de recomendação^(11)^. A análise minuciosa dos artigos, na íntegra, permitiu reconhecer de que forma os estudos selecionados abordam o desenvolvimento de práticas voltadas ao alcance do ODS 11, sendo essas organizadas em categorias.

## RESULTADOS

O cruzamento dos descritores resultou em 2.379 estudos. Após, realizou-se a leitura dos títulos e resumos dos trabalhos encontrados. Foram selecionados 95 artigos para a realização de leitura flutuante, sendo que 11 não se encontravam disponíveis *online* e 50 não atenderam aos critérios de inclusão, restando 34 para serem triados.

A segunda etapa da seleção correspondeu à leitura na íntegra dos 34 documentos completos, dos quais 26 não responderam ao questionamento do estudo e três artigos estavam duplicados. Dessa forma, o *corpus* final constituiu em uma amostra de cinco artigos. Esses estudos foram salvos em uma pasta, utilizando um código (ex., A1, sendo A de artigo, e 1, o número da ordem). A Figura 1 apresenta o fluxograma do processo de seleção dos artigos.

**Figura 1:** Fluxograma representativo da seleção dos estudos analisados nesta pesquisa. Crato, Ceará, Brasil, 2023

Entre os artigos selecionados, observa-se uma discreta predominância de estudos internacionais, sendo dois do Brasil^(12,13)^, um da Eslovênia^(14)^ e dois da Espanha^(15,16)^, com contribuições práticas de enfermeiros relacionadas ao ODS 11. O idioma mais prevalente foi o inglês, presente em quatro^(12,14-16)^ dos artigos, sendo que, em apenas um^(13)^, foi utilizado o português. Os artigos foram publicados entre os anos de 2011 e 2021, com predominância nos últimos cinco anos^(12,15,16)^. Apesar de os ODS surgirem no ano de 2015, trabalho^(13)^ publicado no ano de 2011 trouxe noções que culminaram com o pensamento do ODS 11, por trazer a figura do enfermeiro como um ator importante no desempenho de ações de cuidado coletivo para a saúde dos indivíduos. Isso reforça a decisão de não utilizar recorte temporal no estudo, pois, apesar de os ODS terem sido publicados no ano de 2015, os ODM, anteriores aos ODS já abordavam questões ambientais.

Todos os artigos selecionados estão indexados na base de dados *Web of Science*, sendo que dois estão publicados no periódico *Sustainability* ^(14,16)^, um, no *Globalization and Health* ^(15)^, um, no *International Journal of Health Promotion and Education* ^(12)^, e um, na Revista Latino-Americana de Enfermagem^(13)^. No que tange ao delineamento dos estudos, dois se tratam de estudos qualitativos^(12,13)^, dois, estudos de caso^(14,15)^, e um, estudo descritivo com análise quantitativa^(16)^.

Os estudos elencados trouxeram ações que podem ser desenvolvidas no âmbito das cidades e comunidades, buscando torná-las espaços mais saudáveis e sustentáveis. Alguns trabalhos trazem o enfermeiro como figura relevante nesses processos, contudo outros estudos o trouxeram como parte integrante dos diversos personagens envolvidos nessa empreitada, estando, nesse caso, o seu papel diluído entre as estratégias desenvolvidas pelos mais variados setores da sociedade.

Portanto, todos os artigos selecionados neste estudo abordam a execução do ODS 11 a partir de uma visão coletiva e territorial da sua aplicação, ressaltando a importância do envolvimento dos gestores, de profissionais de diferentes setores e dos mais diversos personagens da sociedade no alcance desse objetivo, o que pode ser constatado no Quadro 2.

**Quadro 2:** Características dos estudos selecionados quanto à autoria, ano, país, objetivo do estudo, delineamento, principais achados, nível de evidência e grau de recomendação, 2023

Variáveis	Artigos selecionados
**Código de identificação do artigo**	**A1^(^** ^12)^
**Autor/ano/país**	Almeida ACL, Davey P. / (2020)/ Brasil.
**Título do artigo**	*Integrating health promotion into sustainable development goal 11: major challenges and learned lessons from Healthy Municipalities, Cities and Communities (HMC) in Brazil*
**Objetivos do estudo**	Examinar como as iniciativas de tornar cidades e municípios saudáveis podem contribuir para integrar a promoção da saúde na implementação do ODS 11, criando sinergias nos níveis locais. Propor uma estrutura para integrar a promoção da saúde na implementação do ODS 11 em nível local.
**Delineamento**	Pesquisa qualitativa, utilizando entrevista como fonte de coleta de dados.
**Principais achados**	Desenvolvimento de projetos de educação e desenvolvimento local, com foco na formação de gestores locais e autonomia comunitária. A participação social é pilar da promoção da saúde e ferramenta essencial para a implementação do ODS 11. O enfermeiro aparece entre os personagens que atuam para essa finalidade.
**Nível de evidência/grau de recomendação**	4/C
**Código de identificação do artigo**	**A2** ^(13)^
**Autor/ano/país**	Fortuna CN, Matumoto S, Pereira MJB, Mishima SM, Kawata LS, Camargo-Borges C./ (2011)/ Brasil
**Título do artigo**	O enfermeiro e as práticas de cuidados coletivos na estratégia saúde da família
**Objetivos do estudo**	Identificar e analisar as práticas de cuidados coletivos do enfermeiro na estratégia saúde da família e seus conhecimentos estruturantes.
**Delineamento**	Pesquisa qualitativa, utilizando-se entrevistas semiestruturadas como fonte de coleta de dados.
**Principais achados**	O enfermeiro é o ator importante no desencadeamento das ações de cuidado coletivo na saúde da família, pois propõe, organiza, desenvolve e avalia tais ações. A ruptura com o modo dominante e instituído de intervir sobre o território de saúde carece de dispositivos que interroguem sua lógica e exponham seu funcionamento, desnaturalizando e desestabilizando seus arranjos e significados, possibilitando, assim, a construção de novas práticas em saúde e novos modos de produção de subjetividade.
**Nível de evidência/grau de recomendação**	4/C
**Código de identificação do artigo**	**A3** ^(14)^
**Autor/ano/país**	Szander N, Ros-McDonell L, Fuente-Aragone MV, Vodopivec R./ (2018)/ Eslovênia.
**Título do artigo**	*Sustainable Urban Homecare Delivery with Different Means of Transport*
**Objetivos do estudo**	Comparar diferentes meios de transporte em ambiente urbano como entrada crucial de um algoritmo para agendamento de atividades de assistência domiciliar, tendo como prioridade máxima a satisfação do paciente.
**Delineamento**	Estudo de caso.
**Principais achados**	Rotas eficientes e confiáveis que considerem as metas de sustentabilidade urbana, reduzindo o tempo de deslocamento e a jornada de trabalho de enfermeiros que atuam na assistência domiciliar quando utilizado transporte público, bicicleta e/ou caminhada.
**Nível de evidência /grau de recomendação**	4/C
**Código de identificação do artigo**	**A4** ^(15)^
**Autor/ano/país**	Rubio OR, Daher C, Fanjul G, Gascon M, Mueller N, Pajín L, Plasencia A, Rueda DR, Thondoo M, Nieuwenhuijsen MJ/ (2019)/ Espanha.
**Título do artigo**	*Urban health: an example of a “health in all policies” approach in the context of SDGs implementation*
**Objetivos do estudo**	Compreender as ligações entre determinantes sociais da saúde, exposições ambientais, comportamento, resultados de saúde e políticas urbanas dentro dos ODS, seguindo uma lógica *health in all policies* (HiAP); revisar e analisar os elementos-chave de uma abordagem HiAP como um acelerador dos ODS no contexto do planejamento urbano e de transporte; e descrever as lições aprendidas com a implementação prática das ações integradas em saúde em cidades da Europa, África e América Latina.
**Delineamento**	Estudo de caso com implementação de ferramenta estratégica de avaliação.
**Principais achados**	A análise confirmou que a estrutura dos ODS oferece uma oportunidade para formular e implementar políticas com uma abordagem HiAP. Três aspectos importantes são destacados: 1) a importância do trabalho intersetorial e da equidade em saúde como uma questão transversal nas ações de desenvolvimento sustentável, entrando as intervenções de profissionais de saúde, como os enfermeiros; 2) coerência das políticas, governança da saúde e participação das partes interessadas como questões-chave; e 3) a necessidade de dados de alta qualidade.
**Nível de evidência/grau de recomendação**	4/C
**Código de identificação do artigo**	**A5** ^(16)^
**Autor/ano/país**	Córdoba PJ, Esteban VA, Bento B./ (2021)/ Espanha.
**Título do artigo**	*The Commitment of Spanish Local Governments to Sustainable Development Goal 11 from a Multivariate Perspective*
**Objetivos do estudo**	Analisar o compromisso dos governos locais espanhóis com o ODS-11, indicando possíveis melhorias com o objetivo de cumprir a Agenda 2030.
**Delineamento**	Estudo descritivo de natureza quantitativa, utilizando a técnica de estudo X-STATIS para análise dos dados.
**Principais achados**	Os resultados indicam uma tendência positiva para o cumprimento do ODS-11, em que os governos progressistas se preocupam com questões de urbanização inclusiva e sustentável como resultado de uma maior participação cidadã, e governos conservadores se concentram na melhoria e segurança de favelas em espaços públicos inclusivos e acessíveis. Nesse cenário, profissionais de saúde, como enfermeiros, aparecem como coadjuvantes no desenvolvimento de ações que contribuam para o desenvolvimento inclusivo e sustentável.
**Nível de evidência/grau de recomendação**	4/C

## DISCUSSÃO

As discussões sobre o impacto das mudanças que ocorreram no *meio ambiente* têm sido mais frequentes nos últimos tempos, apresentando indícios de sua relevância para os ecossistemas, independentemente de seu tamanho ou escala. No caso dos seres humanos, a escala das populações, a repercussão e a magnitude das alterações ambientais causadas pelas espécies têm feito, dessa questão, uma prioridade na busca pela preservação da sobrevivência do planeta^(17)^.

É perceptível que o número de artigos selecionados, para este estudo, foi pouco expressivo, e isso se deve ao fato de existirem poucas publicações que contemplem contribuições de enfermeiros para a melhoria de espaços públicos saudáveis e sustentáveis, o que pode sinalizar para a necessidade urgente de maior atuação por parte da enfermagem na saúde ambiental, considerando que o profissional enfermeiro atua, principalmente, na promoção da saúde e prevenção de doenças, e a maioria dessas perpassa por questões ambientais.

A seguir, serão apresentados os tópicos desta discussão, elaborados a partir dos artigos incluídos neste estudo, nomeadamente: 1) Desenvolvimento de projetos de educação com foco na formação de gestores locais e autonomia comunitária; 2) Participação social e promoção da saúde para a implementação do Objetivo de Desenvolvimento Sustentável 11; 3) O enfermeiro como facilitador de ações de cuidado coletivo na saúde da família; 4) Construção de novas práticas em saúde e novos modos de produção de subjetividade; 5) Utilização de transporte público, bicicleta e/ou caminhada em cidades sustentáveis.

### 1) Desenvolvimento de projetos de educação com foco na formação de gestores locais e autonomia comunitária

Entre os 17 objetivos, o ODS 3 (Boa saúde e bem-estar) é considerado relevante para o alcance de outros objetivos. O ODS 11 (Cidades e comunidades sustentáveis) é um objetivo fundamental a ser incluído nos níveis locais no que diz respeito à consecução dos ODS. Foi possível identificar que os municípios estão desenvolvendo projetos de educação de desenvolvimento local, com foco na formação de gestores locais e autonomia comunitária, para a segurança alimentar e promoção da saúde, prevenção de violência contra mulheres com foco na inclusão social, empoderamento feminino e autoestima^(12)^. Essas ações estão sendo desenvolvidas, principalmente, no âmbito dos serviços da Atenção Primária à Saúde (APS), na qual o enfermeiro ocupa um papel de liderança na organização e implementação desses projetos.

Entretanto, a Constituição Federal do Brasil, em seu Artigo 182, estabeleceu que o município é responsável pelo planejamento e execução da política urbana. Isso estabelece que os planos diretores sejam obrigatórios para municípios com mais de 20.000 habitantes^(18)^, bem como procedimentos para planejamento urbano participativo, incluindo a aprovação pela Câmara Municipal antes da implementação. De fato, trazendo os principais temas discutidos pela comunidade, as iniciativas das Cidades e Municípios Saudáveis (CMS) direcionam os governos locais para a inclusão da promoção da saúde nos planos diretores participativos^(12)^.

Nesse sentido, é possível concluir que a falta de apoio do governo federal dificulta a implantação ampla das CMS no Brasil, e muito provavelmente dificultou a disponibilidade de infraestrutura da promoção da saúde para garantir aos gestores locais a implementação dos projetos das CMS, incluindo o nível de sensibilização, recursos humanos e financiamento^(12)^. Da mesma forma, ainda existe o empecilho pelo fato de que, mesmo que alguns sanitaristas reconheçam a convergência da saúde e da promoção do bem-estar nas cidades, a saúde pública apresenta resistência em adotar a promoção da saúde como paradigma norteador, e as políticas públicas de saúde ainda estão centradas nas doenças^(19)^.

Em particular, o compromisso dos governos locais com o desenvolvimento sustentável das cidades é demonstrado pela urbanização de favelas, aumento do acesso à moradia, fornecimento de serviços básicos adequados aos cidadãos (Habitação adequada) e aumento da sustentabilidade nos sistemas de transporte (Viajantes). Além disso, a participação direta da sociedade civil aumentou nos últimos anos (Participação cidadã), assim como a preocupação com os países menos desenvolvidos, para os quais a assistência financeira aumentou (Transferências). Também houve aumento da proporção média de superfície pavimentada em cidades com espaços livres de uso público (via pública), o que foi acompanhado por uma melhora na qualidade do ar, além de redução dos níveis de partículas de menor diâmetro (poluição do ar) que causam a maioria das doenças respiratórias, o que, em geral, melhora a vida urbana^(16)^.

Contudo, ainda há muito o que se fazer em relação à educação para o desenvolvimento de espaços públicos saudáveis e sustentáveis. Portanto, são necessários maiores investimentos por parte dos governos locais para a concretização de modelos educativos, possibilitando uma melhor infraestrutura e capacitação dos recursos humanos para uma verdadeira sensibilização da sociedade, bem como para que se atinja uma conscientização solidária que vise à preservação do bem público e da vida como um todo.

### 2) Participação social e promoção da saúde para a implementação do Objetivo de Desenvolvimento Saudável 11

A participação social e a integração dos ODS nos planos diretores foram identificadas como fatores-chave que podem promover a implementação integrada do ODS 11 nos níveis locais^(12)^. Quanto aos desafios a serem superados, a falta de uma estratégia nacional com as diretrizes gerais para implantação nos municípios apoiada pelo governo federal e a ausência de avaliação da efetividade dos resultados alcançados pelas CMS foram apontadas pelas partes interessadas como os aspectos mais importantes que podem dificultar a implementação dos ODS no nível local^(20-22)^.

De acordo com a ONU, a implementação dos ODS deve ser baseada em uma abordagem participativa^(23)^. De fato, a participação social é um pilar principal da estratégia das CMS, aprimorando o processo e as mudanças nos níveis locais^(21)^. As iniciativas das CMS envolvem colaboração e participação de diferentes atores e o uso de estratégias participativas distintas. Além disso, a mobilização e a participação coletiva nos processos de tomada de decisão são princípios da promoção da saúde^(19)^. A APS pode ser uma das formas efetivas de disseminar à sociedade esses princípios basilares que unem a saúde à sustentabilidade, colocando o enfermeiro e sua equipe como peças-chave desse processo de sensibilização.

A vontade política, a participação da comunidade e o treinamento desses recursos humanos são fatores facilitadores que viabilizam a implementação das iniciativas das CMS^(24)^, tendo a participação da comunidade como parte essencial da promoção da saúde eficaz^(25)^. A adoção de métodos participativos (ex., avaliações participativas, planejamento participativo, desenvolvimento de redes virtuais, ferramentas de governo eletrônico e projetos colaborativos), bem como de ações intersetoriais em projetos e iniciativas locais pode fortalecer a participação da comunidade, facilitando uma implementação mais integrada e eficiente do ODS 11 em nível local^(12)^. As APS, por exemplo, deveriam receber mais investimentos por parte dos governos locais para criarem mais canais participativos juntos à comunidade, visando uma maior integração desta no desenvolvimento de espaços urbanos sustentáveis.

Os governos progressistas, favoráveis ao aumento dos gastos por meio do endividamento, concentram seus esforços na urbanização inclusiva e sustentável, a partir de uma maior participação cidadã, que leva à construção de trechos pavimentados e ao desenvolvimento de novas áreas residenciais^(16)^. Por outro lado, governos conservadores priorizam a melhoria de favelas, espaços públicos seguros, inclusivos e acessíveis e a importância da coleta diária de resíduos sólidos urbanos. Além disso, a ideologia parece estar perdendo influência em outros alvos, como os relacionados às mudanças climáticas^(16)^.

### 3) Enfermeiro como facilitador de ações de cuidado coletivo na saúde da família

As ações de cuidado no modo hegemônico de fazer saúde carecem de estratégias para mudança, pois ainda estão voltadas para o indivíduo, sendo curativas e desarticuladas das reais necessidades de saúde^(26)^. Nessa perspectiva teórica, o coletivo é considerado composição múltipla de várias conexões, potências e rupturas^(27)^. São fluxos de vida e de desejo que se entrecortam com barragens que os interrompem; são interconexões cambiantes que se singularizam em encontros e desencontros de afetos, modos de viver e valores^(27)^.

Para fins de pesquisa, consideraram-se ações coletivas de cuidado em saúde aquelas comumente aceitas nesse campo, como a construção conjunta do diagnóstico da área (processo de territorialização), atividades de promoção da saúde, de participação e controle social, de educação em saúde e ações intersetoriais^(13)^.

No estudo de Fortuna *et al*.^(13)^, um enfermeiro falou que ações de cuidados coletivos são aquelas que se planeja, avalia e discute com a equipe, implementando-as com o intuito de: buscar a aceitação e a adesão da população, e que essa tenha mais facilidade na vida e qualidade de vida, que seria adoecer menos; ter mais oportunidades, uma vida mais digna, um bom emprego, uma boa moradia, instruções de higiene básica; planejar a vida, não só em relação aos filhos, mas aos gastos, entendendo o fato de que as doenças não são só aquilo que parecem; e ter mais acesso à informação e aos cuidados gerais que a unidade pode e deve oferecer.

Embora ainda incipiente, no conjunto das falas dos enfermeiros, essa perspectiva pode ser potencializadora de outros modos de fazer e saber cuidados coletivos, pois convoca uma complexidade para as necessidades de saúde e para o processo de trabalho, para a qual se fazem necessárias a interlocução de saberes e a corresponsabilização entre equipe, família e outros setores^(13)^.

Destarte, o enfermeiro surge com um papel de destaque no âmbito dos serviços de saúde, principalmente de nível primário, no que concerne à reflexão sobre as novas formas de se fazer saúde, de uma maneira mais sustentável e em harmonia com o meio, incorporando, junto à equipe multiprofissional, ações que viabilizem atingir o ODS 11. O desenvolvimento urbano tem um impacto significativo na saúde física e mental e na habitação das pessoas, sendo que problemas de ordem ambiental podem influenciar para que as cidades se tornem epicentros de doenças não transmissíveis, sobrecarregando os sistemas de saúde^(28,29)^.

### 4) Construção de novas práticas em saúde e novos modos de produção de subjetividade

As mudanças sem precedentes nas últimas décadas levaram a um aumento na complexidade das estruturas sociais, problemas globais de saúde e desigualdades dentro e entre as nações. Os desafios das mudanças climáticas e as transições epidemiológicas e demográficas que levam ao aumento de doenças não transmissíveis e ao envelhecimento da população exigem reformular o modo como desenvolvemos políticas públicas voltadas à saúde^(30)^. As cidades abrigam mais da metade da população mundial^(31)^, e o contexto urbano oferece uma oportunidade inédita para entender os vínculos entre a saúde, seus determinantes sociais e o meio ambiente, e como implementar soluções seguindo uma abordagem intersetorial^(15)^.

Uma compreensão mais profunda das interligações na forma como as cidades são projetadas, planejadas, construídas e governadas e como isso afeta diretamente a saúde humana evoluiu significativamente nos últimos anos. Dois marcos globais impulsionaram a ideia de que os processos locais de tomada de decisão que reconhecem as políticas urbanas são, de fato, as principais intervenções de saúde pública. O primeiro é a aprovação, em 2015, da Agenda 2030 para o Desenvolvimento Sustentável^(32)^, composta por 17 ODS e 169 metas, com abrangência geográfica global.

O segundo marco ocorreu em 2016, com a recém-adotada Nova Agenda Urbana na Habitat III, a Conferência das Nações Unidas sobre Habitação e Desenvolvimento Urbano Sustentável^(31)^. Esta foi a primeira vez que a ‘saúde’ apareceu como uma questão transversal e foi explicitamente reconhecida como um componente central do planejamento urbano e da governança, além da prestação de serviços de saúde. A OMS reforçou esses vínculos reunindo evidências científicas cada vez maiores que conectam a qualidade do *design* urbano e do transporte com uma variedade de resultados de saúde^(33)^. Por exemplo, há evidências científicas o suficiente para vincular o estilo de vida e os hábitos alimentares a resultados de saúde como obesidade e diabetes^(34)^, poluição do ar com doenças cardiovasculares, respiratórias e cânceres^(35)^, ou poluição sonora com problemas de saúde mental e doenças cardiovasculares^(36)^.

Espaços abertos verdes e azuis dentro e ao redor das cidades são muito importantes. A preservação das bacias hidrográficas reduz a contaminação da água potável, economizando nos custos de purificação da água. Reciclar, reutilizar e reduzir os resíduos sólidos elimina a necessidade de queimá-los ou enterrá-los, melhorando a qualidade do ar, reduzindo a contaminação da água e do solo. Uma melhor gestão de águas residuais e esgotos, em um contexto de aumento das temperaturas e eventos climáticos extremos relacionados à mudança climática, também melhora a saúde pública, reduzindo a exposição à água e doenças transmitidas por mosquitos, como as recentes epidemias urbanas de Zika e Chikungunya^(37)^.

A OMS define HiAP como “uma abordagem das políticas públicas em todos os setores que sistematicamente leva em consideração as implicações das decisões para a saúde, busca sinergias e evita impactos nocivos à saúde, a fim de melhorar a saúde da população e a equidade em saúde”^(15,37)^. A estratégia HiAP fornece uma “governança horizontal” forte e eficaz^(38)^, com uma abordagem para problemas de saúde complexos que envolvem os mais altos níveis de governo, liderança política e executiva, levando à definição de prioridades efetivas, inovação na formulação de políticas e implementação de soluções sustentáveis^(15)^.

Por fim, um aspecto fundamental da implementação dos ODS de saúde urbana é garantir que o compromisso de não deixar ninguém para trás seja traduzido em ação efetiva. Isso requer uma compreensão precisa das populações-alvo, suas necessidades e circunstâncias, e ninguém melhor que o enfermeiro para atuar nessa perspectiva, bem como através da educação em saúde, capacitando a população em relação a essas questões ambientais e salutares para o futuro das próximas gerações. É importante, também, que a informação disponível seja desagregada de acordo com os principais eixos de desigualdades, como classe social, gênero, idade ou etnia/migração, utilizando ferramentas e métricas apropriadas que devem estar amplamente disponíveis e incluem o Índice de Saúde Urbana, que fornece informações sobre desigualdades de saúde em pequenas áreas dentro das cidades, ou a Ferramenta de Avaliação e Resposta para a Equidade na Saúde Urbana^(39)^, que mede e toma medidas para combater as desigualdades.

No entanto, as informações necessárias para medir as desigualdades socioeconômicas de forma eficiente (exposição ou dados de resultados por diferentes grupos sociais no nível de pequena área ou, idealmente, georreferenciados) geralmente não estão disponíveis, principalmente em locais com recursos mais baixos.

### 5) Utilização de transporte público, bicicleta e/ou caminhada em cidades sustentáveis

As metas do ODS 11 referem-se a elementos prejudiciais, como a poluição do ar, mas também a exposições ambientais positivas, como espaços verdes abertos. O ODS 11.2 refere-se ao “acesso a sistemas de transporte seguros, baratos, acessíveis e sustentáveis para todos”, notadamente expandindo o transporte público, e, embora não explicitamente mencionado nos ODS, promovendo o ciclismo e a caminhada^(15)^. A adoção dessas formas de transporte pode ser estimulada pelos profissionais de saúde, principalmente pelo enfermeiro, através da educação em saúde nas consultas de enfermagem, incentivando hábitos mais saudáveis que corroborarão espaços públicos verdadeiramente sustentáveis.

Esses elementos também são dependentes entre si e, portanto, suscetíveis a mudanças como consequência do planejamento urbano e das políticas e intervenções de transporte. De fato, um crescente corpo de evidências científicas sobre os impactos das políticas urbanas na saúde pode esclarecer os riscos e informar a tomada de decisão para o desenvolvimento sustentável^(40)^. Políticas urbanas saudáveis podem reduzir significativamente as doenças infecciosas e não transmissíveis e melhorar o bem-estar. Por exemplo, o *design* urbano compacto capitaliza a densidade populacional para reduzir as emissões de gases de efeito estufa e melhorar a mobilidade, a capacidade de caminhar e a coesão social e, portanto, a saúde e o bem-estar^(41)^.

O transporte público eficiente, em combinação com redes de ciclismo, promove mais atividade física, diminuindo a poluição do ar e reduzindo as mortes e lesões no trânsito em geral^(42)^. Os resultados de saúde são bons mobilizadores de políticas de outros setores que podem ser impopulares, como restrições de tráfego ou limites de velocidade nas cidades. Vários vieses são comumente apresentados no planejamento urbano e de transporte, como na área da mobilidade, em que a maioria das intervenções é baseada nas necessidades e perspectivas das pessoas saudáveis, ricas e do sexo masculino^(43,44)^. Processos participativos se tornam necessários para identificar prioridades inclusivas em subpopulações vulneráveis (mulheres, idosos, portadores de deficiência).

Uma vez que a utilização predominante de veículos motorizados nas cidades continuará a ter um forte impacto ambiental e socioeconômico^(45)^, a crescente pressão sobre as realidades urbanas tem levado a um interesse muito maior por todos os aspectos da sustentabilidade no planejamento urbano, seu desenvolvimento e todos os tipos de transporte (bens, serviços e pessoas). Atualmente, existe muita preocupação em desenvolver políticas, programas e projetos que levem em consideração a sustentabilidade^(46)^.

Estudo denotou a redução no total de horas de trabalho dos enfermeiros por dia pela combinação de ônibus e caminhada, mostrando que os enfermeiros que andam de bicicleta elétrica reduziram, respectivamente, cerca de 35% a 44% do tempo total que os enfermeiros passariam viajando. Esse resultado é aplicável a um ambiente urbano, onde a rede de transporte público é suficiente e o ciclismo é permitido em um número razoável de vias^(14)^. Uma melhor gestão da Atenção Domiciliar pode apoiar o uso eficiente dos recursos das instituições de saúde, a Atenção Domiciliar de alta qualidade e as aspirações a comunidades habitáveis e ao desenvolvimento sustentável. Atualmente, existe uma tendência geral de tornar os serviços urbanos mais sustentáveis, especialmente em áreas urbanas mais densas.

### Limitações do estudo

Nesta revisão, foram incluídos cinco artigos, devido à escassez de pesquisas realizadas neste âmbito. Levando-se em consideração que a temática do ODS 11, embora seja atual e de grande relevância para as cidades e comunidades urbanas, tenha uma necessidade de maiores incentivos à realização de estudos por parte dos pesquisadores em saúde ambiental, a mesma visa à mudança de paradigmas e criação de propostas educacionais que venham redefinir os modos de vida da população não só no país, mas em todo o planeta. Recomenda-se a realização de novos estudos com delineamentos que possuam maior nível de evidência.

### Contribuições para as áreas da enfermagem, saúde ou políticas públicas

Este estudo proporciona uma reflexão profunda acerca das ações, as quais o enfermeiro pode ser um forte aliado junto à equipe multiprofissional, desenvolvendo-as com a finalidade de melhorar ou transformar espaços públicos em ambientes mais saudáveis e sustentáveis, já que se trata de um dos objetivos da Agenda 2030, tendo a sociedade, como um todo, envolvida nesse processo. Nesse sentido, o enfermeiro aparece como um grande facilitador de práticas que contribuem para esse cenário, e ele precisa reconhecer esse espaço de liderança que possui para poder dar início às estratégias que viabilizem essa transformação. Logicamente, o enfermeiro não estará sozinho nessa empreitada, pois deve contar com as parcerias intersetoriais e governamentais para alcançar esse objetivo tão almejado.

## CONSIDERAÇÕES FINAIS

Para desenvolver as ações elencadas neste estudo, os enfermeiros precisam de apoio intersetorial e maior investimento por parte dos governos locais para poder elaborar ferramentas com a finalidade de promoção do educar e do cuidar, para uma real sustentabilidade, de modo a favorecer o envolvimento dos sujeitos no processo educativo, contribuir para a participação cidadã e desenvolver a autonomia dos envolvidos dentro do processo de construção de espaços saudáveis. Busca-se que, por meio de uma educação baseada em problemas, sejam utilizadas as experiências do cotidiano profissional, de forma sistemática, estabelecidas em uma perspectiva dialógica e participativa. Espera-se que, partindo de uma realidade conduzida por reflexão crítica, chegue-se a uma ação transformadora.
